# Rotenone activates the LKB1-AMPK-ULK1 signaling pathway to induce autophagy and apoptosis in rat thoracic aortic endothelial cells

**DOI:** 10.1186/s40360-024-00755-5

**Published:** 2024-05-23

**Authors:** Xiaoyu Chang, Zeyuan Li, Mi Tian, Ziwei Deng, Lingqin Zhu, Guanghua Li

**Affiliations:** 1 School of Public Health, Ningxia Medical University, Yinchuan, 750004 China; 2 School of Basic Medical Sciences, Ningxia Medical University, Yinchuan, 750004 China

**Keywords:** Rotenone, Autophagy, Apoptosis, LKB1-AMPK-ULK1

## Abstract

**Background:**

The specific mechanism by which rotenone impacts thoracic aortic autophagy and apoptosis is unknown. We aimed to investigate the regulatory effects of rotenone on autophagy and apoptosis in rat thoracic aortic endothelial cells (RTAEC) via activation of the LKB1-AMPK-ULK1 signaling pathway and to elucidate the molecular mechanisms of rotenone on autophagy and apoptosis in vascular endothelial cells.

**Methods:**

In vivo, 60 male SD rats were randomly selected and divided into 5 groups: control (Con), DMSO, 1, 2, and 4 mg/kg groups, respectively. After 28 days of treatment, histopathological and ultrastructural changes in each group were observed using HE and transmission electron microscopy; Autophagy, apoptosis, and LKB1-AMPK-ULK1 pathway-related proteins were detected by Western blot; Apoptosis levels in the thoracic aorta were detected by TUNEL. In vitro, RTAEC were cultured and divided into control (Con), DMSO, 20, 100, 500, and 1000 nM groups. After 24 h of intervention, autophagy, apoptosis, and LKB1-AMPK-ULK1 pathway-related factors were detected by Western blot and qRT-PCR; Flow cytometry to detect apoptosis levels; Autophagy was inhibited with 3-MA and CQ to detect apoptosis levels, and changes in autophagy, apoptosis, and downstream factors were detected by the AMPK inhibitor CC intervention.

**Results:**

Gavage in SD rats for 28 days, some degree of damage was observed in the thoracic aorta and heart of the rotenone group, as well as the appearance of autophagic vesicles was observed in the thoracic aorta. TUNEL analysis revealed higher apoptosis in the rotenone group’s thoracic aorta; RTAEC cultured in vitro, after 24 h of rotenone intervention, showed increased ROS production and significantly decreased ATP production. The flow cytometry data suggested an increase in the number of apoptotic RTAEC. The thoracic aorta and RTAEC in the rotenone group displayed elevated levels of autophagy and apoptosis, and the LKB1-AMPK-ULK1 pathway proteins were activated and expressed at higher levels. Apoptosis and autophagy were both suppressed by the autophagy inhibitors 3-MA and CQ. The AMPK inhibitor CC reduced autophagy and apoptosis in RTAEC and suppressed the production of the AMPK downstream factors ULK1 and P-ULK1.

**Conclusions:**

Rotenone may promote autophagy in the thoracic aorta and RTAEC by activating the LKB1-AMPK-ULK1 signaling pathway, thereby inducing apoptosis.

## Background

Rotenone has been a common insecticide used in agriculture for over a century due to its high efficiency, low toxicity, and safety properties [1]. As people become increasingly concerned about food safety, the potential for development and research expands. Studies have shown that pesticide exposures are likely to stretch far beyond the occupational setting, and many individuals who are not professionally exposed to pesticides may be unwittingly exposed [2]. Rotenone is highly lipophilic, freely crosses cell membranes, and readily crosses the blood-brain barrier, thus readily producing systemic effects on mitochondria [3]. Currently, domestic and foreign researchers studying rotenone have primarily focused on the induced damage of rotenone on the central nervous system. Among these, the rotenone-induced Parkinson’s disease model and its pathogenic mechanism have received extensive research. However, research on rotenone-induced vascular injury and its impact on the incidence of cardiovascular diseases is limited.

Previous studies have shown that rotenone-mediated mitochondrial dysfunction is associated with cardiovascular diseases [4]. According to research, neointima formation increases mitochondrial activity, glycolysis, and respiration, all of which can encourage the production of reactive oxygen species and cell migration and proliferation [5–7]. Rotenone may also serve a beneficial effect in high-fat diet animals by reducing mitochondrial division and ROS generation, consequently limiting neointima formation and smooth muscle migration following arterial damage and preventing the progression of atherosclerosis [8]. And rotenone has recently been demonstrated to protect kidneys from oxidative stress and inflammation and improve renal function at the level of chronic kidney disease [9]. Rotenone has also been found to suppress tumor progression by affecting autophagy flux and inhibiting cancer cell proliferation, such as lung and liver cancer [10, 11].

Autophagy is a highly conserved catabolic process that may avoid cell damage, promote cell survival in the face of energy or nutrient shortages, and react to various cytotoxic damage [12]. The interaction between autophagy and apoptosis is complicated. Both pathways are regulated by common factors, they share similar components, and each can regulate and modify the activity of the other pathway. An obvious puzzle is that autophagy plays a role in both cytoprotection and cell death [13]. Autophagy and apoptosis are regulated by each other. Autophagy plays a cytoprotective role in response to most forms of cellular stress. For example, increasing cell death at the level of autophagy is a very viable therapeutic target for many diseases [14]. Activation of the Akt/mTOR pathway has been shown in studies to initiate autophagy and protect against rotenone-induced cell damage in SH-SY5Y cells [15]. Rotenone significantly downregulated the phosphorylated active form of PI3K/Akt/mTOR pathway and autophagy marker Beclin1 in the substantia nigra, inhibiting autophagic flux and caspase3-mediated apoptosis before causing cell death [16]. These findings suggest that autophagy is a critical scavenger in the deleterious effects of toxic rotenone in the clusters made by rotenone-treated cells [17]. However, in some instances where autophagy is over-regulated (e.g., Beclin-1 is overexpressed in mammalian cells and ULK1 is overexpressed in Drosophila), autophagy promotes cell death by activating apoptosis.

In this study, the effects of rotenone on autophagy and apoptosis of thoracic aorta and thoracic aorta endothelial cells (RTAEC) in rats were studied through the intervention of rotenone at different concentrations, and the molecular mechanism of rotenone on autophagy and apoptosis of normal vascular endothelial cells was clarified. It provides a theoretical basis for rotenone’s toxicological and pharmacological effects on the cardiovascular system, as well as a reference for scientific experimental research on its medicinal dosage.

## Methods

### Experimental animals and grouping

In the experiment, 60 male SD rats were randomly divided into 5 groups: control group (Con), DMSO (solvent control) group, and rotenone group (according to the preliminary experimental results, the rotenone exposure dose was set as 1, 2 and 4 mg·kg^−1^·d^−1^, with 12 rats in each group. The experimental rats were administered by gavage for 28 consecutive days. The control group was given normal saline (0.5 ml/100 g) and the DMSO group was given the same gavage volume of DMSO at a concentration of 20%. After the experiment, the rats were anesthetized by intraperitoneal injection of pentobarbital sodium at a dose of 30 mg/kg, and the samples were collected.

### Cell culture and rotenone treatment

The cells were incubated in DMEM high sugar medium supplemented with 10% fetal bovine serum with Penicillin-Streptomycin Solution at 37 °C in a constant temperature incubator with 5% CO_2_. The rotenone group was given different concentrations of rotenone intervention (20, 100, 500, and 1000 nM) for 24 h. Autophagy inhibitors 3-MA and CQ were applied to cells 3 and 4 h before rotenone intervention, respectively, and then treated with rotenone for 24 h. AMPK inhibitor CC (Dorsomorphin/Compound C, CC) interfered with cells 2 h before rotenone treatment

### Reagents

RTAEC cell lines was purchased from Shanghai Saiqi Bioengineering Co. Ltd. Rotenone was purchased from Sigma. Hematoxylin-eosin staining kit was purchased from Tiangen Biotech (Beijing) Co, Ltd (China). The TUNEL kit was purchased from UE Landi Biotechnology (China); the DAPI-containing blocker was purchased from Solarbio Biotechnology (China). RNA extraction kit, reverse transcription reagent, and cDNA synthesis kit were purchased from Takara Biomedical Technology (Beijing) Co, Ltd. Beclin1 and Bax were purchased from Proteintech Group. AMPK, P-AMPK, P62, and Cleaved-Caspase3 were purchased from Cell Signaling Technology. P-ULK1 purchased from Bioss. LC3 and Caspase3 were purchased from Abcam. Bcl-2 was purchased from Bioworld Technology. 3-MA, CQ, and CC were purchased from Medchemexpress.

### Preparation of paraffin sections and HE staining

The fresh rat thoracic aorta was taken and fixed in 4% paraformaldehyde solution, rinsed with tap water for 2 h, and then stored in 70% alcohol overnight. The next day, the tissues were dehydrated successively in gradient ethanol. Take out the tissue embedding box from anhydrous ethanol and put it into containers I and II containing fresh xylene for 30 min respectively. Waxing, embedding, sectioning, dewaxing, dehydration, nuclear staining in hematoxylin solution, differentiation in 0.5% differentiation solution, eosin staining, dropping neutral resin, and film sealing for subsequent film observation.

### Transmission electron microscopy (TEM)

The thoracic aortas were dissected into 1.0 mm^3^-sized sample blocks, prefixed with 2.5% glutaraldehyde and post-fixed with 1% osmic acid, then washed with phosphate-buffered saline (PBS), post-treated with graded acetone, and post-embedded in tissue cells were infiltrated with tissue embedding agent, and then stained with uranyl acetate and lead citrate before drying for observation of the ultrastructure. Finally, TEM was used to observe and collect images under a magnification of 5000×, and 25,000×.

### TUNEL assay

Paraffin slices were dewaxed and dehydrated, washed with pure water, and incubated with 100 μl proteinase K dropwise for 20 min at room temperature. The positive tablets were prepared after PBS washing. Add 100 μl TUNEL balanced buffer to each sample, incubate for 5 min, and then add 50 μl TUNEL reaction mixture. Place the sample flat in a wet box, and incubate it at 37 °C for 2 h in the dark; the samples were washed twice by immersion in PBS for 5 min/time. Then 0.1% Triton X-100 with 5 mg/mL Bovine Serum Albumin in buffer solution was prepared with PBS for washing the samples, immersed in pure water for 5 min, then put into gradient alcohol for 5 min, immersed in anhydrous ethanol for 5 min, finally in fresh xylene for transparent treatment, and then use an anti-fluorescence quenching sealing solution to remove bubbles to make the sealing film complete.

### ROS measurement

RTAEC were cultured in six-well plates and exposed to different concentrations of rotenone for 24 h. After 24 h the cells were collected and washed twice and the dichlorodihydrofluorofluorescein diacetate (DCFH-DA) probe (1:1000) was added to the cells and incubated for 20 min washed twice with PBS, centrifuged, and resuspended in PBS. Detection by fluorescent enzyme marker. The ROS content of the control group was set as 1, and the data of each group was normalized.

### ATP measurement

The culture medium was discarded and 200 μl of lysis solution was added to each of the 6 well plates, followed by repeated blowing to lyse the cells. After lysis, the cells were centrifuged at 4 °C for 5 min at 12,000 g. The supernatant was taken in a black 96-well plate and assayed for ATP using a Multifunctional Enzyme Analyzer. The ATP content of the control group was set as 1, and the data of each group was normalized.

### Flow cytometry

Collect RTAEC and wash twice with cold PBS; dilute 10X binding buffer with pure water to 1X binding buffer and then suspend the cells with 400 μl of 1X Annexin V binding buffer at 1 × 10^6^ cells/ml; add 5 μl of Annexin V-Alexa Fluor 488 staining solution to the cell suspension. Fluor 488 staining solution was added to the cell suspension, gently mixed, and incubated for 15 min at 4 °C in a refrigerator protected from light, then 8 μl of PI staining solution was added and gently mixed and protected from light, and incubated for 5 min at 4 °C in a refrigerator.

### Western blotting (WB)

The proteins were denatured and then electrophoresed on polyacrylamide gels with a loading volume of 40 μg protein. The proteins were transferred to PVDF membranes and then closed in 5% skimmed milk for 2 h at room temperature, followed by the addition of primary antibodies. Incubate PVDF membranes at 4 °C overnight. After washing 3 times with TBST (5 min/time), a secondary antibody (1:10,000) was added and incubated for 1 hour, then wash 3 times with TBST (5 min/time). Blots were developed with ECL detection reagents.

### Quantitative reverse transcription-polymerase chain reaction (qRT-PCR)

Extract RNA according to the kit instructions, use a fluorescence spectrophotometer to measure the optical density values at wavelengths of 260 and 280 nm, and calculate the purity and concentration of RNA. Reverse transcription of RNA to cDNA. The qRT-PCR reaction conditions were 95 °C pre-denaturation for 30 s; 95 °C denaturations for 5 s and 60 °C annealings and extension for 30 s, a total of 40 cycles. The purity and concentration of RNA were calculated by △△CT method, △CT = CT target gene − CT GAPDH, △△CT = △CT experiment − △CT control, amplification ploidy = 2^−△△CT^. Three sets of technical replicates were set up for each sample. The qRT-PCR primer sequences are listed in Table 1. The CT values for each group were calculated by taking the mean of the three groups as CT values.

**Table 1:** Primer sequences, GenBank accession codes, and expected product sizes

Genes	GenBank accession	Primer sequence (5’-3’)	Length (bp)
GAPDH	NM_017008.4	S:CTGGAGAAACCTGCCAAGTATG	138
		A: GGTGGAAGAATGGGAGTTGCT	
LC3I	NM_199500.2	S: CCTGTCCTGGATAAGACCAAGTT	188
		A: CCGTCTTCATCCTTCTCCTGTT	
LC3II	NM_022867.2	S: TCCGAGAAGACCTTCAAACAGC	233
		A: AAGAAGGCTTGGTTAGCATTGAG	
P62	NM_130405.1	S: TTGAGAAGATTCAGAAGGGAGAGTC	247
		A: TCTTCCTCCTTGGCTTTGTCTC	
Beclin1	NM_001034117.1	S: CTTCAATGCGACCTTCCATATC	258
		A: CCAGAACAGTACAACGGCAACT	
LKB1	M_001108069.1	S: CATCCGACAGATTAGACAGCACA	257
		A: GTCCATTCTGACCCACTTCCTC	
AMPK	NM_023991	S: CACTGGATGCACTCAACACAAC	153
		A: TCACTACCTTCCATTCAAAGTCC	
ULK1	NM_001108341.1	S: ACTGACAGCCTACAGGAGAAACC	126
		A: GGAGCCCACAGTAAATACCACA	

### Statistical analysis

The experimental data were analyzed using SPSS 22.0 and GraphPad Prism 9.3.0 to plot bar charts. All data met normal distribution, and measurement data were expressed as mean ± standard deviation (SD) ($\bar x \pm S$). The one-way ANOVA was used for comparison between multiple groups, and Tukey’s and Dunnett-t-test was used for multiple comparisons between groups.

## Results

### Effect of rotenone on the morphology and structure of rat heart and thoracic aorta

**Fig. 1:**
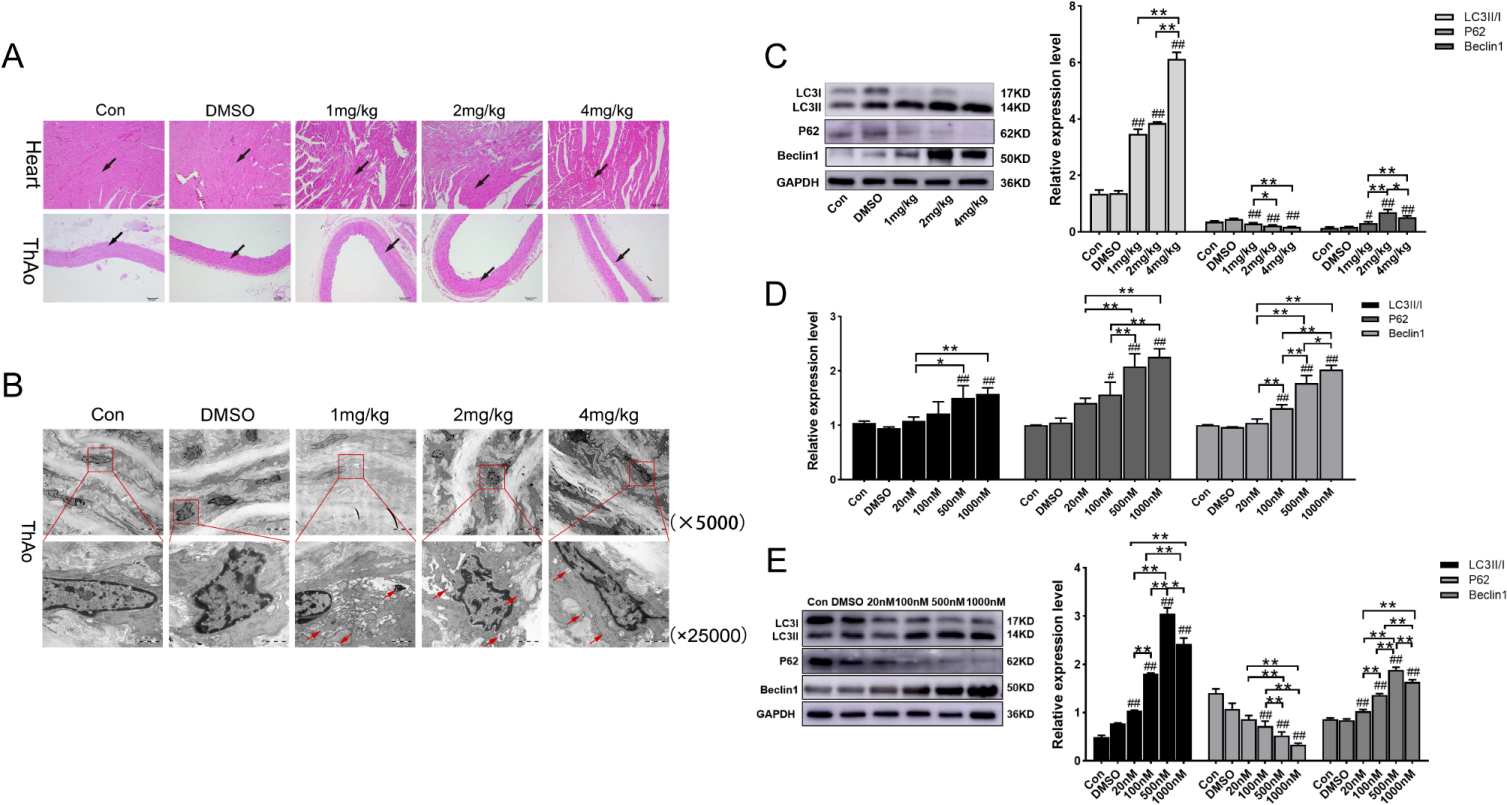
Rotenone induces autophagy in the thoracic aorta and RTAEC. Thoracic aortic autophagy levels were measured in rats 28 days after gavage modeling, and RTAEC was treated for 24 h by exposure to different concentrations of rotenone. **A** Hematoxylin and eosin staining was used for histological examination of the heart and thoracic aorta. The scale bar was 100 µm. **B** Thoracic aortic autophagosomes were visualized by transmission electron microscopy. The scale bars are 5000 µm and 25,000 µm respectively. **C** Western blotting was used to detect LC3, P62, and Beclin1 protein expression. **D** qRT-PCR was used to detect changes in LC3, P62, and Beclin1 genes. **E** Western blotting was used to detect LC3, P62, and Beclin1 protein expression. ThAo stands for the thoracic aorta. The results were expressed as the mean ± SD of three independent experiments. ^#^*P <* 0.05 vs. DMSO; ^##^*P <* 0.01 vs. DMSO; *P stands for *P <* 0.05; **P stands for *P <* 0.01 (Comparison of different doses of rotenone between groups)

The HE staining results showed that, as indicated by the arrow, the myocardium and thoracic aorta in the control and DMSO groups were structurally normal (Fig. 1A). However, the myocardial cells in the rotenone group were vacuolated and degenerated, some myocardial fibers were indistinctly striated, cell morphology was unclear, and myocardial fibers were disorganized and broken. Endothelial cells of the thoracic aorta were absent and exfoliated, and elastic fibers were disorganized and uneven.

### Rotenone promotes autophagy in the thoracic aorta and RTAEC

The transmission electron microscope results showed that the middle membrane of the rotenone treatment group was not stratified clearly and became thinner, with a large amount of collagen infiltrating smooth muscle, and autophagic bodies were found as shown by the red arrow in the figure (Fig. 1B). Western blotting results showed that the expression of thoracic aorta LC3 and Beclin1 in rats in the rotenone group increased compared with the control group; The expression of P62 is significantly reduced (Fig. 1C).

To investigate further the impact of rotenone on RTAEC autophagy, we measured the levels of autophagy-related factors LC3, P62, and Beclin1. The mRNA levels of LC3, P62, and Beclin1 in RTAEC increased significantly at rotenone concentrations of 500 and 1000 nM, while there was no significant change in autophagy levels at rotenone concentrations of 20 nM (Fig. 1D). Results from western blotting revealed that the rotenone group’s expression of the autophagy-related proteins LC3 and Beclin1 increased significantly when compared to that of the DMSO group, while P62 expression demonstrated a tendency to decline (Fig. 1E). It indicates rotenone induces autophagy in RTAEC.

### Rotenone promotes apoptosis in the thoracic aorta and RTAEC

**Fig. 2:**
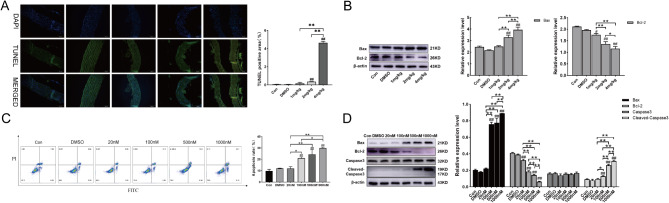
Rotenone induces apoptosis in the thoracic aorta and RTAEC. Thoracic aortic apoptosis levels were measured in rats 28 days after gavage modeling, and RTAEC was treated for 24 h by exposure to different concentrations of rotenone. **A** TUNEL assay of the number of apoptotic cells in the thoracic aorta. The scale bar is 50 μm. **B** Western blotting was used to detect Bax and Bcl-2 protein expression. **C** Flow detection of apoptosis by Annexin V/PI. **D** Western blotting was used to detect Bax, Bcl-2, Caspase3, and Cleaved-Caspase3 protein expression. The results were expressed as the mean ± SD of three independent experiments. ^#^*P <* 0.05 vs. DMSO; ^##^*P <* 0.01 vs. DMSO; *P stands for *P <* 0.05; **P stands for *P <* 0.01 (Comparison of different doses of rotenone between groups)

Detection of apoptosis by TUNEL assay revealed an increase in the number of apoptotic cells in the thoracic aorta of the rotenone-treated group and an increased incidence of apoptosis in the 2 and 4 mg/kg groups compared to the DMSO group (Fig. 2A). Similarly, Western blotting results showed a concentration-dependent pro-apoptotic occurrence of rotenone, as evidenced by increased expression of pro-apoptotic Bax and decreased expression of the apoptosis-inhibiting protein Bcl-2 (Fig. 2B). It indicates that rotenone can promote apoptosis.

To detect the effect of different concentrations of rotenone on apoptosis. Firstly, the apoptosis was detected by Annexin V/PI flow cytometry, the graph shows Annexin-FITC signal as the horizontal coordinate and PI signal as the vertical coordinate, with normal living cells in the lower left quadrant, early apoptotic cells in the lower right quadrant, mid-late apoptosis in the upper right quadrant, and necrotic cells in the upper left quadrant. The apoptosis rate is the sum of the upper right quadrant and lower right quadrant percentages. The apoptosis was significantly increased at the concentrations of 100, 500, and 1000 nM of rotenone, and there was no significant change in apoptosis at the concentration of 20 nM (Fig. 2C); Western blotting results also showed that rotenone promoted apoptosis, showing an increase in Bax and Cleaved-Caspase3 expression and a decrease in Bcl-2 expression with increasing rotenone concentration (Fig. 2D).

### Effect of different concentrations of rotenone on ROS and ATP levels in RTAEC

**Fig. 3:**
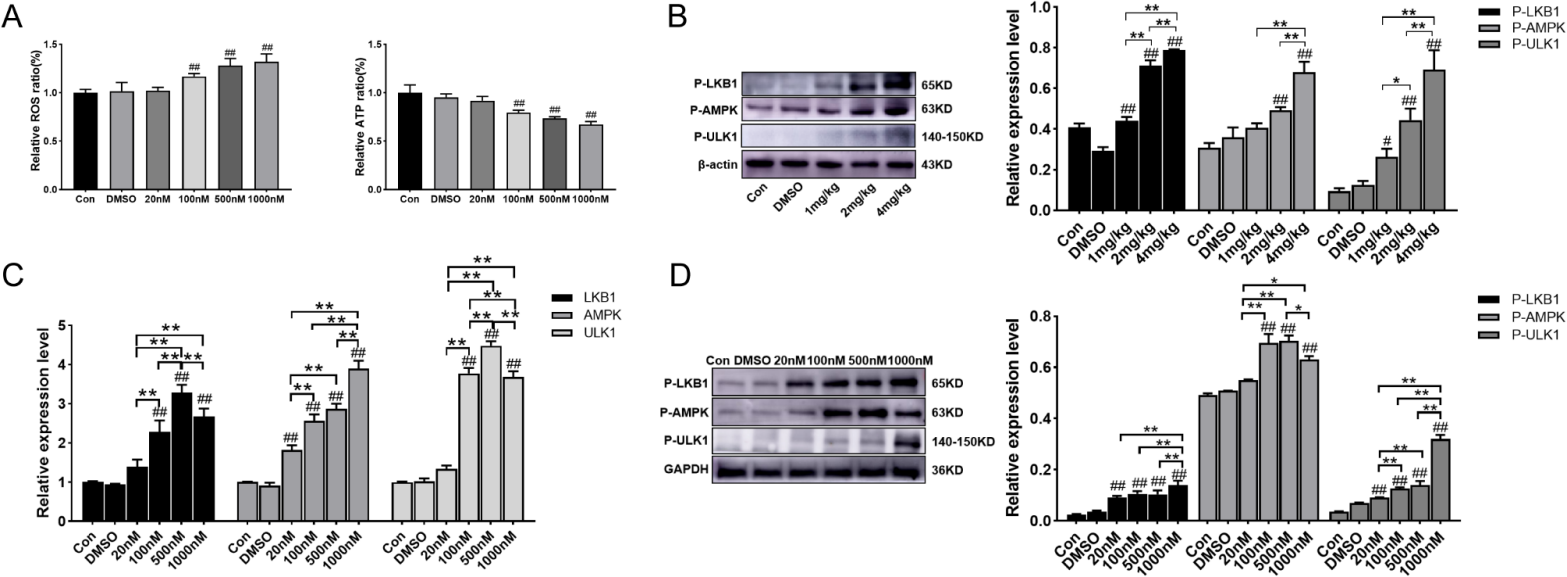
Rotenone activates the LKB1-AMPK-ULK1 signaling pathway in the thoracic aorta and RTAEC. Thoracic aortic autophagy levels were measured in rats 28 days after gavage modeling, and RTAEC was treated for 24 h by exposure to different concentrations of rotenone. **A** Detection of ROS and ATP levels in RTAEC. **B** Western blotting was used to detect P-LKB1, P-AMPK, and P-ULK1 protein expression in the thoracic aorta. **C** qRT-PCR was used to detect changes in LKB1, AMPK, and ULK1 genes. **D** Western blotting was used to detect the expression of P-LKB1, P-AMPK, and P-ULK1 proteins. The results were expressed as the mean ± SD of three independent experiments. ^#^*P <* 0.05 vs. DMSO; ^##^*P <* 0.01 vs. DMSO; *P stands for *P <* 0.05; **P stands for *P <* 0.01 (Comparison of different doses of rotenone between groups)

The levels of ROS and ATP in RTAEC treated with different concentrations of rotenone were examined. The results showed that the levels of ROS in different rotenone intervention groups were directly proportional to the concentration of rotenone, while the levels of ATP were inversely proportional to it. That is, as the concentration of rotenone intervention in RTAEC increased (Fig. 3A), the expression level of ROS increased, while the production of ATP decreased significantly.

### Rotenone activates the LKB1-AMPK-ULK1 pathway in the thoracic aorta and RTAEC

The effect of rotenone on the LKB1-AMPK-ULK1 signaling pathway in the thoracic aorta was examined, and the results showed that rotenone activated the signaling pathway in a concentration-dependent manner, and rotenone significantly increased the protein expression of P-LKB1, P-AMPK and P-ULK1 (Fig. 3B). After 24 h of rotenone intervention, the activation status of the LKB1-AMPK-ULK1 signaling pathway was detected and verified by qRT-PCR, which revealed that at the mRNA level LKB1, AMPK, and ULK1 were all significantly upregulated compared to the DMSO group (Fig. 3C). Activation of the LKB1-AMPK-ULK1 signaling pathway was also shown at the protein level, as evidenced by a significant increase in the expression of P-LKB1, P-AMPK, and P-ULK1 proteins (Fig. 3D). All of these results indicated that rotenone activated the LKB1-AMPK-ULK1 pathway in RTAEC.

### Autophagy inhibitors 3-MA and CQ suppress autophagy and apoptosis in RTAECs

**Fig. 4:**
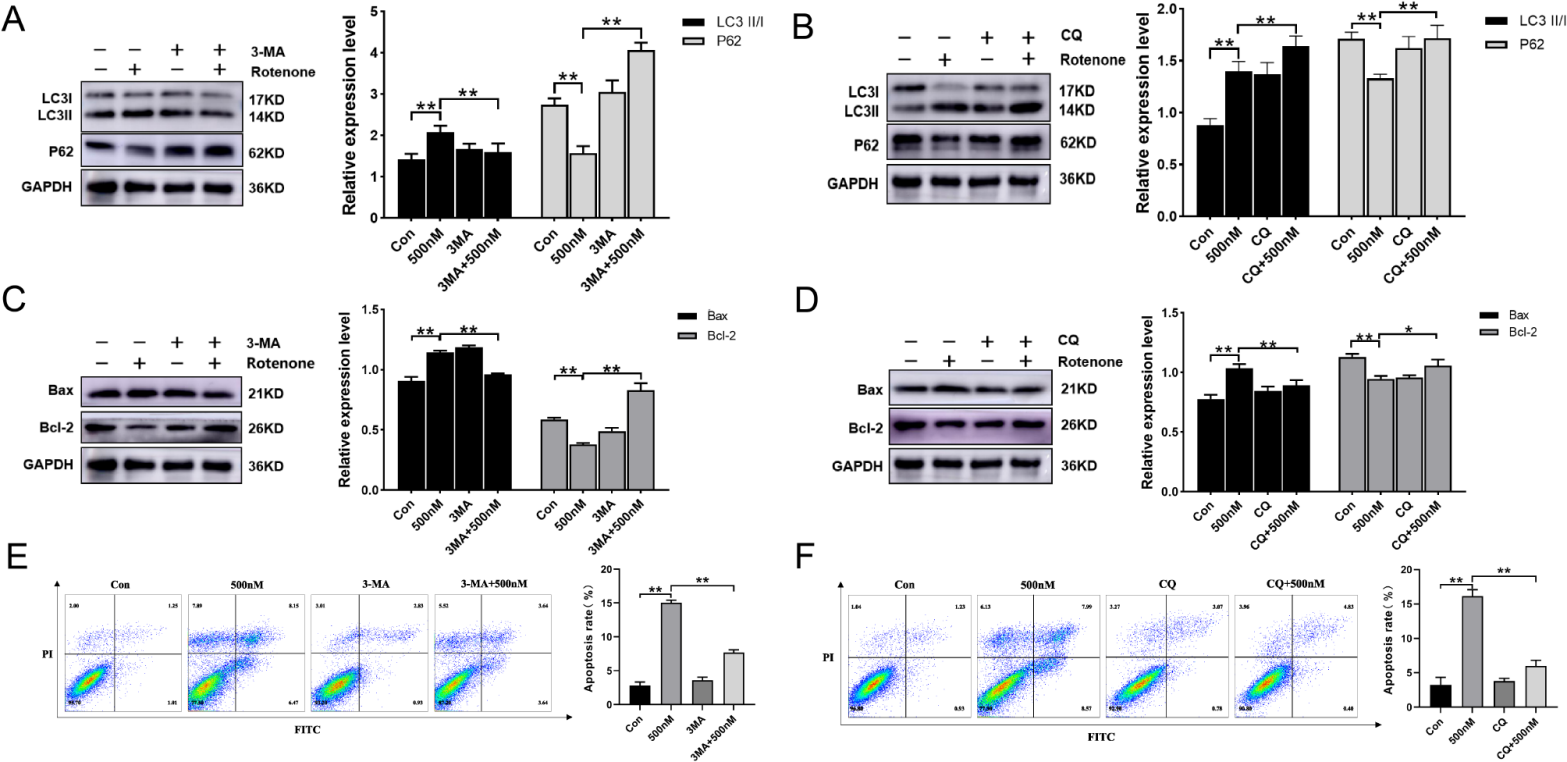
3-MA and CQ inhibit RTAEC autophagy and apoptosis. RTAEC were treated with 5 mM of 3-MA for 3 h and 10 μM of CQ for 4 h, followed by exposure to 500 nM rotenone for 24 h. **A** Western blotting was used to detect the expression of LC3 and P62 proteins in RTAEC after 3-MA treatment. **B** Western blottingwas used to detect the expression of LC3 and P62 proteins in RTAEC after CQ treatment. **C** Western blotting was used to detect the expression of Bax and Bcl-2 proteins in RTAEC after 3-MA treatment. **D** Western blotting was used to detect the expression of Bax and Bcl-2 proteins in RTAEC after CQ treatment. **E** The apoptosis of RTAEC treated with 3-MA was detected by flow cytometry. **F** The apoptosis of RTAEC treated with CQ was detected by flow cytometry. The results were expressed as the mean ± SD of three independent experiments. *P stands for *P <* 0.05; **P stands for *P <* 0.01.

The autophagy inhibitors 3-MA and CQ were used to verify whether RTAEC autophagy levels were reversed after rotenone intervention, and it was found that 3-MA and CQ did inhibit rotenone-mediated autophagy (Fig. 4). LC3 expression was decreased and P62 expression was increased after the 3-MA intervention compared to the 500 nM group (Fig. 4A), while both LC3 and P62 expression was up-regulated after CQ intervention (Fig. 4B). 3-MA and CQ were further used to verify the relationship between autophagy and apoptosis. It was found that inhibition of autophagy was followed by a reversal of apoptosis levels, indicating that apoptosis was mediated by autophagy. The results showed that cellular Bax expression increased and Bcl-2 expression decreased after 500 nM rotenone treatment compared with the control group. However, protein expression levels were reversed to some extent after 3-MA and CQ intervention (Fig. 4C, D). Flow cytometry also showed a decrease in apoptosis after 3-MA and CQ intervention (Fig. 4E, F).

### The AMPK inhibitor CC blocks the LKB1-AMPK-ULK1 signaling pathway

**Fig. 5:**
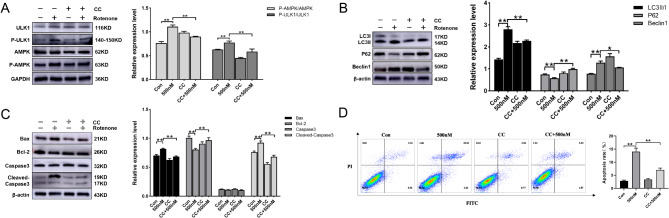
The AMPK inhibitor CC suppresses autophagy and apoptosis in RTAEC. RTAEC were treated with 10 μM CC for 2 h and subsequently exposed to 500 nM rotenone for 24 h. **A** Western blotting was used to detect the expression of P-AMPK/AMPK and P-ULK1/ULK1 proteins in RTAEC after CC treatment. **B** Western blotting was used to detect the expression of LC3, P62, and Beclin1 proteins in RTAEC after CC treatment. **C** Western blotting was used to detect the expression of Bax, Bcl-2, Caspase3, and Cleaved-Caspase3 proteins in RTAEC after CC treatment. **D** The apoptosis of RTAEC treated with CC was detected by flow cytometry. The results were expressed as the mean ± SD of three independent experiments. *P stands for *P <* 0.05; **P stands for *P <* 0.01

To test whether the LKB1-AMPK-ULK1 pathway initiates autophagy causing apoptosis, CC, an AMPK inhibitor, was used to treat rotenone-treated RTAEC. The function of AMPK and its downstream factor ULK1 was discovered to be inhibited by CC. After CC intervention, P-AMPK and P-ULK1 protein expression was significantly decreased when compared to the 500 nM group (Fig. 5A). According to the findings, CC did inhibit the LKB1-AMPK-ULK1 pathway.

### The AMPK inhibitor CC diminishes rotenone-induced autophagy and apoptosis

To determine whether autophagy mediating apoptosis is triggered by the LKB1-AMPK-ULK1 pathway, CC, an inhibitor of AMPK, was selected to intervene in rotenone-treated RTAEC. The expression of RTAEC autophagy-related proteins LC3 and Beclin1 was lower in the 500 nM + CC group after CC intervention compared to the 500 nM group, whereas P62 expression was increased (Fig. 5B). Compared with the 500 nM group, the expressions of apoptosis-related proteins Bax and Cleaved-caspase3 were significantly decreased in the 500 nM + CC group, while the expression of Bcl-2 was increased (Fig. 5C), which indicated that the apoptosis level was decreased after CC inhibited autophagy of the LKB1-AMPK-ULK1 pathway. This finding is consistent with flow cytometry (Fig. 5D). These results suggest that autophagy does indeed trigger apoptosis through the LKB1-AMPK-ULK1 pathway.

## Discussion

Rotenone is a mitochondrial complex I inhibitor that impairs the proper functioning of energy homeostasis. The oxidative respiratory chain’s ability to transport electrons can be hampered by damage to or inhibition of complex I, which can have systemic consequences on the body. With the increase of rotenone exposure in recent years, the research on rotenone has become more and more thorough, but the research on the effect of rotenone on arterial endothelial cells is very limited. Therefore, it is very critical to study the effect of rotenone on blood vessels and endothelial cells.

The thoracic aorta is the backbone of the arterial system and plays an important role in the circulation of the system. Endothelial cells, as the first layer of cells lining blood vessels, have direct contact not only with blood components but also with endocrine organs [18]. Vascular endothelial cells can also sense hemodynamic changes and blood signals [19], and react by releasing vasoactive substances. Therefore, the importance of endothelial cells to human health cannot be overemphasized.

The autophagy process contributes to the removal of damaged organelles, protein aggregates, and macromolecules, as well as their recycling and the maintenance of cellular homeostasis [20]. Beclin1 is a core role in autophagy and constitutes a molecular platform that regulates the formation and maturation of autophagy [21]. LC3 is one of the important markers of autophagy flux, and the detection of LC3 transformation (LC3-I to LC3-II) has become a reliable method to monitor autophagy. However, since both autophagy induction and autophagy flux blocking can increase the ratio of LC3II/LC31, ubiquitin-binding protein (Sequestosomel, P62) is also required to comprehensively analyze autophagy levels to distinguish between the two conditions. P62 is an important protein linking LC3II with ubiquitinated substrates and can be degraded in autophagic lysosomes [22]. In the study, LC3, P62, and Beclin1 were used to characterize autophagy and it was found that at the animal and cellular levels, our results showed an increased LC3-II/LC3-I ratio and a decreased P62, indicating that autophagic flow is smooth. However, it is interesting to note that P62 expression was increased in the PCR results, contrary to the Western bloting results. This may be due to the influence of complex cross-feedback regulatory mechanisms, because during cellular autophagy, P62 proteins may be affected by complex regulatory mechanisms, including the regulation of transcriptional levels and protein stability, among others. Therefore, WB and PCR may detect regulatory changes at different levels, leading to inconsistent results. But proteins are the main bearers of life’s activities.TEM also observed the presence of autophagosomes. In conclusion, rotenone promotes autophagy.

Apoptosis is the process by which cells stop growing and dividing, ultimately leading to programmed cell death. The initiation of apoptosis depends on the activation of a series of cysteine aspartate proteases (Caspases) [23]. Anti-apoptotic Bcl-2 family proteins [24–26] and members of the pro-apoptotic protein family have been implicated as cellular variants that control the threshold of cell death in mammalian cells [27–29]. Mitochondria release cytochrome C, which then activates effector caspase3 [30], which is negatively regulated by a family of apoptosis-inhibitory proteins [31]. Bax and Bak are central effectors of apoptosis, leading to the permeability of the outer mitochondrial membrane and triggering apoptosis. After rotenone treatment, the expression of pro-apoptotic protein increased, while the expression of anti-apoptotic protein decreased. The results of TUNEL staining and flow cytometry also indicated that rotenone intervention significantly increased the level of apoptosis in a concentration-dependent manner. All these results confirmed that rotenone can promote cell apoptosis.

Treatment of RTAEC with rotenone revealed an increase in ROS production, a decrease in ATP production, and an increase in AMP: ATP ratio. Liver kinase B1 (LKB1) and its direct target substrate AMP-activated protein kinase (AMPK) form an integral signaling axis that senses the depletion of intracellular ATP levels [32]. When nutrient/energy deficits occur in the body, LKB1 and AMPK are activated as P-LKB1 and P-AMPK. The increase of ROS also activated LKBI, leading to the up-regulation of P-LKB1. Among autophagy-related proteins, ULK1 is the most upstream component of the core autophagy machinery and is essential for the initiation of autophagy [33]. AMPK binds to and directly phosphorylates ULK1/Atg1, and this phosphorylation-dependent modification of ULK1 is essential for the induction of autophagy [34]. It has been shown that treatment with rotenone leads to the activation of AMPK and phosphorylation of ULK1 in a feedforward manner.

**Fig. 6:**
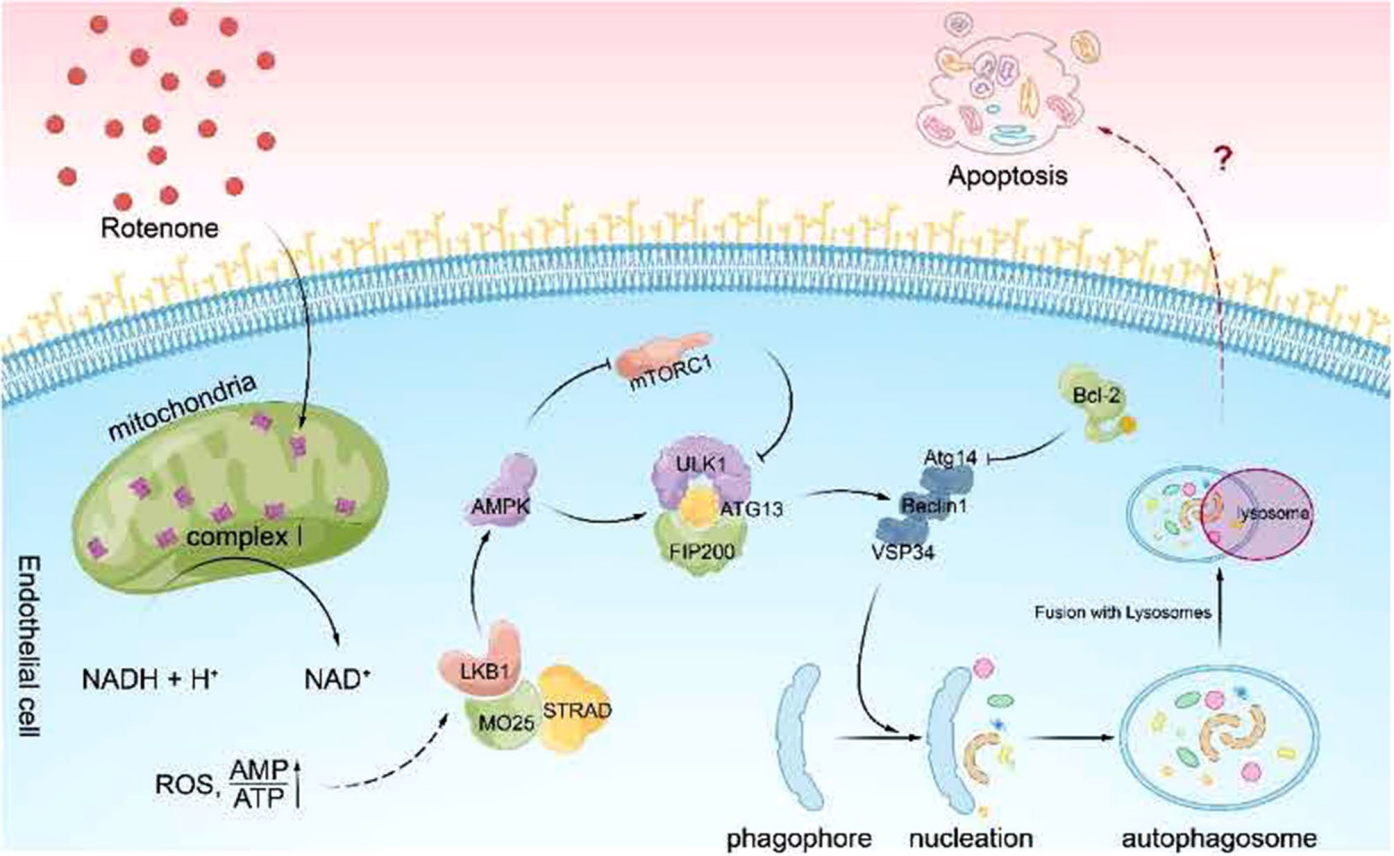
Schematic diagram of the research hypothesis

This is consistent with the results of the present study, in which the LKB1-AMPK-ULK1 pathway was activated and expression was increased in rotenone-treated thoracic aorta and RTAEC compared with the DMSO group. To clarify the relationship between rotenone-induced autophagy and apoptosis, autophagy, and apoptosis levels were downregulated synchronously after the intervention of autophagy inhibitors 3-MA and CQ, indicating that autophagy promoted the occurrence of apoptosis. To further clarify whether the LKB1-AMPK-ULK1 pathway promotes apoptosis by activating autophagy, RTAEC was intervened with the AMPK inhibitor CC, and the results showed that the expression of P-AMPK and P-ULK1 was significantly reduced. And after inhibition of AMPK, autophagy, and apoptosis levels were downregulated simultaneously. All these results indicated that rotenone intervention in RTAEC activated the LKB1-AMPK-ULK1 signaling pathway to induce the onset of autophagy and further promote apoptosis (Fig. 6).

In terms of the functional impact of autophagy, on the one hand, autophagy protects cells and has therapeutic effects. In some cases of uncontrolled upregulation of autophagy, the autophagy protein Beclin1 is overexpressed in mammalian cells, and Atg1/ULK1 is overexpressed in Drosophila), autophagy may lead to cell death, possibly by activating apoptosis. Autophagy and apoptosis are two crucial self-destructive processes that maintain cellular homeostasis [13], which is characterized by their morphology and regulated through signal transduction mechanisms [35]. In this study, autophagy and apoptosis were likewise found to act synergistically, that is, the intervention of rotenone on RTAEC induced both autophagy and promoted apoptosis, while the latter might be caused by excessive autophagy, that is, cytotoxic autophagy of cells leading to apoptosis [36–38].

There may be a “switch” between autophagy and apoptosis, which has important implications for the prevention and treatment of cardiovascular diseases and other diseases, as well as for drug development. It has been reported [39] that the Bcl-2/Beclin1 complex may act as a “transfer switch” to determine the fate of cells. Beclin1 can bind to Bcl-2 through its BH3 structural domain to separate Bcl-2 from pro-apoptotic proteins, thus activating apoptosis. This is similar to the results of this study, Beclin1 protein expression increased and Bcl-2 expression decreased after rotenone intervention with RTAEC. Therefore, the occurrence of apoptosis in this study may be related to the pro-apoptotic “switch” of the Bcl-2/Beclin1 complex. However, the specific mechanism needs further exploration.

## Conclusion

In summary, the present study provides a novel insight that rotenone, as a mitochondrial complex I inhibitor, induces increased cellular ROS production and increased AMP/ATP ratio, which activates the LKB1-AMPK-ULK1 signaling pathway triggering excessive autophagy leading to eventual apoptosis. This is different from autophagy in the traditional sense, which mainly refers to protective autophagy [40], whereas autophagy in this study promotes apoptosis and acts synergistically with apoptosis to jointly influence cell fate.

## Data Availability

The datasets used and/or analyzed during the current study are available from the corresponding author upon reasonable request.

## Abbreviations

HE: Hematoxylin-Eosin
CQ: Chloroquine
CC: Dorsomorphin/compound C
3-MA: 3-Methyladenine
DMSO: Dimethyl sulfoxide
LKB1: Liver kinase B1
AMPK: AMP-activated
ULK1: UNC-51-like kinase 1
LC3: Microtubule-associated proteins light chain 3
P62: Nucleoporin 62
Bax: Bcl-2AssaciatedXprotein
Bcl-2: B-cell lymphoma-2
Caspase3: Cysteine aspartate-specific protease 3
Cleaved-Caspase3: Cleaved-Cysteine aspartate-specific protease 3

## References

[1] Xue W, Men S, Liu R. Rotenone restrains the proliferation, motility and epithelial-mesenchymal transition of colon cancer cells and the tumourigenesis in nude mice via PI3K/AKT pathway. Clin Exp Pharmacol Physiol. 2020;47:1484–94. 32282954 10.1111/1440-1681.13320 PMC7384028

[2] Tanner CM, Kamel F, Ross GW, Hoppin JA, Goldman SM, Korell M, et al. Rotenone, paraquat, and Parkinson’s disease. Environ Health Perspect. 2011;119:866–72. 21269927 10.1289/ehp.1002839 PMC3114824

[3] Rekuviene E, Ivanoviene L, Borutaite V, Morkuniene R. Rotenone decreases ischemia-induced injury by inhibiting mitochondrial permeability transition in mature brains. Neurosci Lett. 2017;653:45–50. 28527718 10.1016/j.neulet.2017.05.028

[4] Madiha S, Tabassum S, Batool Z, Liaquat L, Sadir S, Shahzad S, et al. Assessment of gait dynamics in rotenone-induced rat model of Parkinson’s disease by footprint method. Pak J Pharm Sci. 2017;30:943–48. 28655689

[5] Lim S, Lee SY, Seo HH, Ham O, Lee C, Park JH, et al. Regulation of mitochondrial morphology by positive feedback interaction between PKCdelta and Drp1 in vascular smooth muscle cell. J Cell Biochem. 2015;116:648–60. 25399916 10.1002/jcb.25016

[6] Salabei JK, Hill BG. Mitochondrial fission induced by platelet-derived growth factor regulates vascular smooth muscle cell bioenergetics and cell proliferation. Redox Biol. 2013;1:542–51. 24273737 10.1016/j.redox.2013.10.011 PMC3836280

[7] Wang L, Yu T, Lee H, O’Brien DK, Sesaki H, Yoon Y. Decreasing mitochondrial fission diminishes vascular smooth muscle cell migration and ameliorates intimal hyperplasia. Cardiovasc Res. 2015;106:272–83. 25587046 10.1093/cvr/cvv005 PMC4481571

[8] Xia W, Li Y, Wu M, Yin J, Zhang Y, Chen H, et al. Inhibition of mitochondrial activity ameliorates atherosclerosis in ApoE(-/-) mice via suppressing vascular smooth cell activation and macrophage foam cell formation. J Cell Biochem. 2019;120:17767–78. 31131474 10.1002/jcb.29042

[9] Ding W, Xu C, Wang B, Zhang M. Rotenone attenuates renal injury in aldosterone-infused rats by inhibiting oxidative stress, mitochondrial dysfunction, and inflammasome activation. Med Sci Monit. 2015;21:3136–43. 26474533 10.12659/MSM.895945 PMC4614375

[10] Hu W, Tian H, Yue W, Li L, Li S, Gao C, et al. Rotenone induces apoptosis in human lung cancer cells by regulating autophagic flux. IUBMB Life. 2016;68:388–93. 27015848 10.1002/iub.1493

[11] Dikic I, Elazar Z. Mechanism and medical implications of mammalian autophagy. Nat Rev Mol Cell Biol. 2018;19:349–64. 29618831 10.1038/s41580-018-0003-4

[12] Badaboina S, Bai HW, Na YH, Park CH, Kim TH, Lee TH, et al. Novel radiolytic rotenone derivative, rotenoisin B with potent anti-carcinogenic activity in hepatic cancer cells. Int J Mol Sci. 2015;16:16806–15. 26213921 10.3390/ijms160816806 PMC4581171

[13] Prerna K, Dubey VK. Beclin1-mediated interplay between autophagy and apoptosis: new understanding. Int J Biol Macromol. 2022;204:258–73. 35143849 10.1016/j.ijbiomac.2022.02.005

[14] Levine B, Kroemer G. Biological functions of autophagy genes: a disease perspective. Cell. 2019;176:11–42. 30633901 10.1016/j.cell.2018.09.048 PMC6347410

[15] Zhang Q, Zhou J, Shen M, Xu H, Yu S, Cheng Q, et al. Pyrroloquinoline quinone inhibits rotenone-induced microglia inflammation by enhancing autophagy. Molecules. 2020;25. 10.3390/molecules25194359 PMC7582530 32977419

[16] El-Sherbeeny NA, Soliman N, Youssef AM, Abd El-Fadeal NM, El-Abaseri TB, Hashish AA, et al. The protective effect of biochanin A against rotenone-induced neurotoxicity in mice involves enhancing of PI3K/Akt/mTOR signaling and beclin-1 production. Ecotoxicol Environ Saf. 2020;205:111344. 32977283 10.1016/j.ecoenv.2020.111344

[17] Dadakhujaev S, Noh HS, Jung EJ, Cha JY, Baek SM, Ha JH, et al. Autophagy protects the rotenone-induced cell death in alpha-synuclein overexpressing SH-SY5Y cells. Neurosci Lett. 2010;472:47–52. 20117172 10.1016/j.neulet.2010.01.053

[18] Kruger A, Mrowietz C, Lendlein A, Jung F. Interaction of human umbilical vein endothelial cells (HUVEC) with platelets in vitro: influence of platelet concentration and reactivity. Clin Hemorheol Microcirc. 2013;55:111–20. 23445632 10.3233/CH-131695

[19] Mehta D, Malik AB. Signaling mechanisms regulating endothelial permeability. Physiol Rev. 2006;86:279–367. 16371600 10.1152/physrev.00012.2005

[20] Siva Sankar D, Dengjel J. Protein complexes and neighborhoods driving autophagy. Autophagy. 2021;17:2689–705. 33183148 10.1080/15548627.2020.1847461 PMC8526019

[21] Hill SM, Wrobel L, Rubinsztein DC. Correction to: post-translational modifications of Beclin 1 provide multiple strategies for autophagy regulation. Cell Death Differ. 2019;26:2810. 30546075 10.1038/s41418-018-0254-9 PMC6460389

[22] Chung SJ, Nagaraju GP, Nagalingam A, Muniraj N, Kuppusamy P, Walker A, et al ADIPOQ/adiponectin induces cytotoxic autophagy in breast cancer cells through STK11/LKB1-mediated activation of the AMPK-ULK1 axis. Autophagy. 2017;13:1386–403. 28696138 10.1080/15548627.2017.1332565 PMC5584870

[23] Elmore S. Apoptosis: a review of programmed cell death. Toxicol Pathol. 2007;35:495–516. 17562483 10.1080/01926230701320337 PMC2117903

[24] Yang J, Liu X, Bhalla K, Kim CN, Ibrado AM, Cai J, et al Prevention of apoptosis by Bcl-2: release of cytochrome c from mitochondria blocked. Science. 1997;275:1129–32. 9027314 10.1126/science.275.5303.1129

[25] Kharbanda S, Pandey P, Schofield L, Israels S, Roncinske R, Yoshida K, et al Role for Bcl-xL as an inhibitor of cytosolic cytochrome C accumulation in DNA damage-induced apoptosis. Proc Natl Acad Sci U S A. 1997;94:6939–42. 9192670 10.1073/pnas.94.13.6939 PMC21263

[26] Nijhawan D, Fang M, Traer E, Zhong Q, Gao W, Du F, et al Elimination of Mcl-1 is required for the initiation of apoptosis following ultraviolet irradiation. Genes Dev. 2003;17:1475–86. 12783855 10.1101/gad.1093903 PMC196078

[27] Kale J, Osterlund EJ, Andrews DW. BCL-2 family proteins: changing partners in the dance towards death. Cell Death Differ. 2018;25:65–80. 29149100 10.1038/cdd.2017.186 PMC5729540

[28] Adams JM, Cory S. The Bcl-2 protein family: arbiters of cell survival. Science. 1998;281:1322–26. 9735050 10.1126/science.281.5381.1322

[29] Korsmeyer SJ, Shutter JR, Veis DJ, Merry DE, Oltvai ZN. Bcl-2/Bax: a rheostat that regulates an anti-oxidant pathway and cell death. Semin Cancer Biol. 1993;4:327–32. 8142617

[30] Maharjan PS, Bhattarai HK. Singlet oxygen, photodynamic therapy, and mechanisms of cancer cell death. J Oncol. 2022;2022:7211485. 35794980 10.1155/2022/7211485 PMC9252714

[31] Silke J, Meier P. Inhibitor of apoptosis (IAP) proteins-modulators of cell death and inflammation. Cold Spring Harb Perspect Biol. 2013;5. 10.1101/cshperspect.a008730 PMC3552501 23378585

[32] Hardie DG. AMPK–sensing energy while talking to other signaling pathways. Cell Metab. 2014;20:939–52. 25448702 10.1016/j.cmet.2014.09.013 PMC5693325

[33] Zou L, Liao M, Zhen Y, Zhu S, Chen X, Zhang J, et al Autophagy and beyond: unraveling the complexity of UNC-51-like kinase 1 (ULK1) from biological functions to therapeutic implications. Acta Pharm Sin B. 2022;12:3743–82. 36213540 10.1016/j.apsb.2022.06.004 PMC9532564

[34] Paskeh MDA, Asadi A, Mirzaei S, Hashemi M, Entezari M, Raesi R, et al Targeting AMPK signaling in ischemic/reperfusion injury: from molecular mechanism to pharmacological interventions. Cell Signal. 2022;94:110323. 35358642 10.1016/j.cellsig.2022.110323

[35] Gupta R, Ambasta RK, Pravir K. Autophagy and apoptosis cascade: which is more prominent in neuronal death? Cell Mol Life Sci. 2021;78:8001–47. 34741624 10.1007/s00018-021-04004-4 PMC11072037

[36] Gewirtz DA. The four faces of autophagy: implications for cancer therapy. Cancer Res. 2014;74:647–51. 24459182 10.1158/0008-5472.CAN-13-2966

[37] Sharma K, Le N, Alotaibi M, Gewirtz DA. Cytotoxic autophagy in cancer therapy. Int J Mol Sci. 2014;15:10034–51. 24905404 10.3390/ijms150610034 PMC4100138

[38] Gewirtz DA. When cytoprotective autophagy isn’t… and even when it is. Autophagy. 2014;10:391–92. 24419177 10.4161/auto.27719 PMC4077877

[39] Xu HD, Qin ZH. Beclin 1, Bcl-2 and autophagy. Adv Exp Med Biol. 2019;1206:109–26. 31776982 10.1007/978-981-15-0602-4_5

[40] Sun C, Mo M, Wang Y, Yu W, Song C, Wang X, et al Activation of the immunoproteasome protects SH-SY5Y cells from the toxicity of rotenone. Neurotoxicology. 2019;73:112–19. 30904435 10.1016/j.neuro.2019.03.004

